# Deep-learning–enabled spatial frequency domain imaging of the spatiotemporal dynamics of skin physiology

**DOI:** 10.1117/1.JBO.30.4.046008

**Published:** 2025-04-20

**Authors:** Guowu Huang, Yansen Hu, Weihao Lin, Chenfan Shen, Jianmin Yang, Zhineng Xie, Yifan Ge, Xin Jin, Xiafei Qian, Min Xu

**Affiliations:** a The Eighth Affiliated Hospital of Sun Yat-sen University, Department of Equipment, Shenzhen, China; b Wenzhou Medical University, Institute of Lasers and Biomedical Photonics, Biomedical Engineering College, Wenzhou, China; c Hangzhou First People’s Hospital, Chengbei District, Hangzhou, China; d The City University of New York, Hunter College and the Graduate Center, Department of Physics and Astronomy, New York, New York, United States

**Keywords:** optics, spatial frequency domain imaging, real-time imaging, deep learning, skin

## Abstract

**Significance:**

Spatial frequency domain imaging (SFDI) is an emerging optical imaging modality for visualizing tissue absorption and scattering properties. This approach is promising for noninvasive wide field-of-view (FOV) monitoring of biophysiological processes *in vivo*.

**Aim:**

We aim to develop deep-learning–enabled spatial frequency domain imaging (SFDI-net) for real-time large FOV imaging of the optical, structural, and physiological properties and demonstrate its application for probing the spatiotemporal dynamics of skin physiology.

**Approach:**

SFDI-net, based on mapping of a two-layer structure into an equivalent homogeneous medium for spatially modulated light and with a convolutional neural network architecture, produces two-dimensional maps of optical, structural, and physiological parameters for bilayered tissue, including cutaneous hemoglobin concentration, oxygen saturation, scattering properties (reduced scattering coefficient and scattering power), melanin content, surface roughness, and epidermal thickness, with visible spatially modulated light at the camera frame rate.

**Results:**

Compared with traditional approaches, SFDI-net achieves a real-time inversion speed and significantly improves image quality by effectively suppressing noise while preserving tissue structure without oversmoothing. We demonstrate the application of the SFDI-net for monitoring the spatiotemporal dynamics of forearm skin physiology in reactive hyperemia and rhythmic respiration and reveal their intricate patterns in hemodynamics.

**Conclusions:**

Deep-learning–enabled spatial frequency domain imaging and SFDI-net may offer insights into the cardiorespiratory system and have promising clinical utility for disease diagnosis, surveillance, and therapeutic assessment. Future hardware and software advancements will bring SFDI-net to clinical practice.

## 1 Introduction

Alterations in the human skin structure and physiology can serve as valuable indicators of various diseases and pathological conditions within the human body in clinical practice.^1^^–^^3^ Optical imaging has emerged as the preferred modality for skin monitoring, primarily due to its noninvasive nature and remarkable sensitivity to optical property changes associated with physiological and functional variations in the skin.^4^ Spatial frequency domain imaging (SFDI), a label-free and wide field-of-view (FOV) imaging modality sensitive to both the scattering and absorption properties of turbid media, is particularly well suited for comprehensively characterizing skin structure and subcutaneous hemodynamics.^5^^–^^7^ Compared with other optical approaches, SFDI is unique in quantifying tissue absorption and scattering properties over a large field of view while retaining sensitivity to microstructures.^8^^,^^9^ Consequently, SFDI has been used for skin disease monitoring, blood flow quantification, and the assessment of burn wound healing, underscoring its broad potential for clinical applications.^10^^–^^12^ The real-time monitoring of skin structural, physiological, and hemodynamic variations by SFDI across temporal and spatial dimensions holds significant clinical merits for disease diagnosis, surveillance, and therapeutic assessment.^13^

Despite its potential, SFDI encounters challenges primarily associated with information content and reconstruction speed. The leading optical property inversion method calculates a two-dimensional distribution map of optical properties based on least-squares fitting or the lookup table method using a tissue light reflection model based on Monte Carlo simulations or diffusion approximation.^5^^,^^14^ The reconstruction process for producing two-dimensional (2D) images is slow, ranging from 10 s to hours.^15^^,^^16^ Recently, deep learning techniques have been applied to estimate the optical parameters with high spatial resolution, increasing the inversion speed by 300 to 100,000 times.^6^^,^^17^^,^^18^ However, these methods are mostly geared toward recovering tissue absorption and scattering parameters, with limited applicability to broader tissue parameters. The intricacies originating from the layered structure of the skin were further ignored, compromising the fidelity of the recovery.^19^^–^^21^ As a promising alternative, we have previously presented a method for mapping the layered skin structure to a homogeneous medium for SFDI^8^^,^^9^^,^^22^ and recovering a rich set of skin properties, including its optical, structural, and physiological parameters. The perturbative spatial frequency domain imaging (p-SFDI) approach can recover one set of skin property images in 40 s;^23^ however, this approach is insufficient for clinical applications where the ideal imaging frame rate should exceed 10 frames per second (fps).^24^ Therefore, a faster method for recovering optical, structural, and physiological parameters in SFDI needs to be developed to accelerate the translation of SFDI to clinical practice.

This paper presents deep-learning–enabled spatial frequency domain imaging (SFDI-net) for large FOV imaging of the spatiotemporal dynamics of skin physiology. SFDI-net produces 2D maps of the optical, structural, and physiological parameters of the skin, including the cutaneous hemoglobin concentration (THb), oxygen saturation (${\mathrm{StO}}_{2}$), scattering properties [reduced scattering coefficient ($\mu_{s}^{\prime }$) and scattering power (b)], melanin content ($C_{\text{melanin}}$), surface roughness (α), and epidermal thickness (h)], with visible spatially modulated light at the camera frame rate. After briefly reviewing SFDI for layered skin and introducing SFDI-net in Sec. 2, we first demonstrate, through a simulation study and imaging of a melanin nevus, a significant improvement in the robustness and inversion speed of SFDI-net compared with pixel-wise fitting and perturbative SFDI approaches. We then showcase the application of SFDI-net in monitoring the spatiotemporal dynamics of forearm skin physiology during reactive hyperemia and rhythmic respiration. Their intricate patterns in terms of hemodynamic dynamics were revealed by SFDI-net. These findings suggest that deep-learning–enabled spatial frequency domain imaging and SFDI-net may offer novel insights into the cardiorespiratory system and have promising clinical utility for disease diagnosis, surveillance, and therapeutic assessment.

## 2 Materials and Methods

### 2.1 SFDI of Layered Skin

**Fig. 1:**
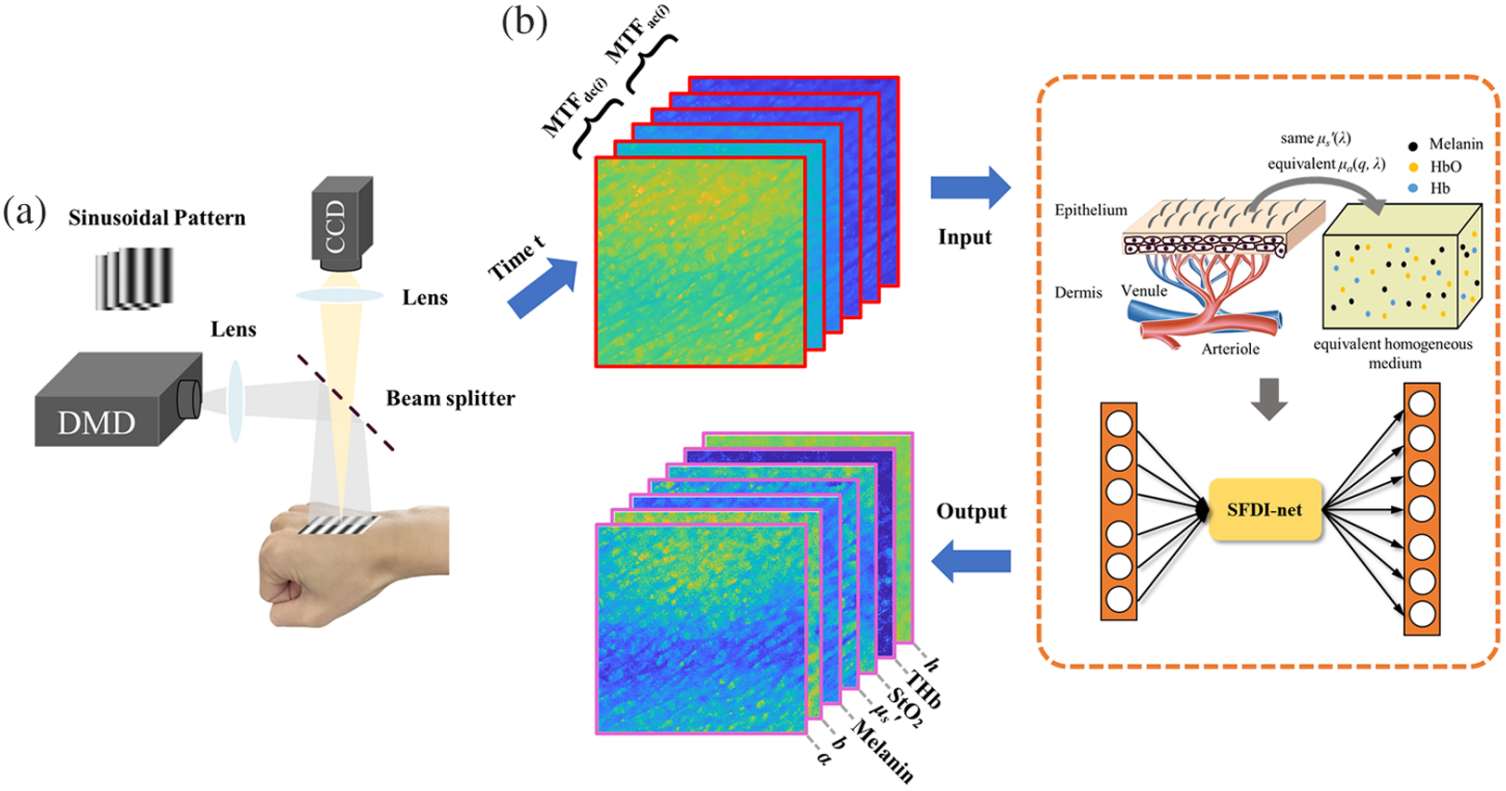
Deep learning enables spatial frequency domain imaging of the spatiotemporal dynamics of skin optical, structural, and physiological parameters with visible spatially modulated light at the camera frame rate. (a) Schematic diagram of the SFDI system. (b) Real-time inversion of the cutaneous hemoglobin concentration (THb), ${\mathrm{StO}}_{2}$, $\mu_{s}^{\prime }$, scattering power (b), melanin content, surface roughness (α), and epidermal thickness (h) by the SFDI-net at time t.

Spatially modulated light illuminates the sample [see Fig. 1(a)]. The illumination patterns are $I_{DC}^{\left(0\right)}+I_{AC}^{\left(0\right)}\;\cos(2\pi f_{x}x+\varphi )$, where $I_{DC}^{\left(0\right)}$ and $I_{AC}^{\left(0\right)}$ are the direct current (DC) and alternating current (AC) amplitudes, respectively; $f_{x}$ is the spatial frequency; x is the coordinate along the x-axis; and φ is the phase.

Diffuse reflectance images are demodulated to extract the direct current intensity ($I_{\mathrm{DC}}$) and the alternating current intensity ($I_{\mathrm{AC}}$).^8^^,^^15^ The conventional three-phase shifting method, widely used for mitigating nonuniform illumination and environmental light effects, is adopted here.^5^ The DC and AC amplitudes are expressed as follows:^25^

$$I_{\mathrm{DC}}=\frac{I_{1}+I_{2}+I_{3}}{3},$$ (1)

$$I_{\mathrm{AC}}=\frac{\sqrt{2}}{3}[(I_{1}-I_{2}{)}^{2}+(I_{2}-I_{3}{)}^{2}+(I_{3}-I_{1}{)}^{2}{]}^{\frac{1}{2}},$$ (2)

from the intensities $I_{1}$, $I_{2}$, and $I_{3}$ of the three images for φ=0,2π/3,4π/3. The modulation transfer function (MTF) is given by

$${\mathrm{MTF}}_{\mathrm{DC}}=\frac{I_{\mathrm{DC}}}{I_{\mathrm{DC}}^{\left(0\right)}},$$ (3)

$${\mathrm{MTF}}_{\mathrm{AC}}=\frac{I_{\mathrm{AC}}}{I_{\mathrm{AC}}^{\left(0\right)}}.$$ (4)

The DC and AC MTFs are subsequently fitted by appropriate light migration models describing photon propagation within the sample to obtain the absorption coefficient $\mu_{a}$ and reduced scattering coefficient $\mu_{s}^{\prime }$.^26^

Biological tissue, such as skin, has a complex layered structure. We have introduced a highly effective approach for imaging layered skin using modulated light.^27^ Layered skin is mapped to a homogeneous turbid medium equivalent in light absorption and scattering for spatially modulated light.^7^^,^^8^ A homogeneous medium has an equivalent absorption coefficient

$$\mu_{a}(q,\lambda )L(q,\lambda )=\mu_{a,\text{epidermis}}(\lambda )h+\mu_{a,\text{dermis}}(\lambda )(L-h),$$ (5)

where $q=2\pi f_{x}$, h is the thickness of the epidermis, and L is the mean probing depth of the structured light

$$L(q,\lambda )=\frac{\int zI(q,z)dz}{\int I(q,z)dz}=\frac{(1+Ql{)}^{2}(2\mu_{t}^{\prime }{)}^{-2}+(1+\mu_{t}^{\prime }l{)}^{2}(2Q{)}^{-2}-2(1+Ql)(1+\mu_{t}^{\prime }l)(Q+\mu_{t}^{\prime }{)}^{-2}}{(1+Ql{)}^{2}(2\mu_{t}^{\prime }{)}^{-1}+(1+\mu_{t}^{\prime }l{)}^{2}(2Q{)}^{-1}-2(1+Ql)(1+\mu_{t}^{\prime }l)(Q+\mu_{t}^{\prime }{)}^{-1}},$$ (6)

where $\mu_{t}^{\prime }\equiv \mu_{a}+\mu_{s}^{\prime }$, $Q\equiv \sqrt{q^{2}+3\mu_{a}(\mu_{a}+\mu_{s}^{\prime })}$, and l is the extrapolation length for diffusing light, which depends on the refractive index mismatch and the roughness of the boundary.^28^ The probing depth of structured light, L, depends on its spatial frequency and wavelength. Equation (5) enforces the equivalence of total light absorption inside the two-layer skin and the mapped homogeneous medium. This strategy of mapping layered skin to an equivalent homogeneous medium has shown efficacy in risk stratification of diabetes and diabetic foot,^7^ noninvasive Sjögren’s syndrome labial salivary gland biopsy,^29^ and coherent hemodynamics spectroscopy of subcutaneous microcirculation.^12^

As the light scattering of the epidermis and dermis is dominated by fractal refractive index fluctuations, the reduced scattering coefficient can be expressed by a power law

$$\mu_{s}^{\prime }(\lambda )=\mu_{s}^{\prime }(540\;\mathrm{nm})(\frac{\lambda }{540\;\mathrm{nm}}{)}^{-b},$$ (7)

where b is the scattering power, and λ is the wavelength in nanometers.^30^^,^^31^ Furthermore, the absorption coefficient for the epidermal and dermal layers can be expressed as

$$\mu_{a,\text{epidermis}}(\lambda )=\varepsilon_{\mathrm{Hb}}(\lambda )C_{\mathrm{Hb}}+\varepsilon_{{\mathrm{HbO}}_{2}}(\lambda )C_{{\mathrm{HbO}}_{2}},$$ (8)

$$\mu_{a,\text{epidermis}}(\lambda )=\varepsilon_{\text{melanin}}(\lambda )C_{\text{melanin}},$$ (9)

where $\varepsilon_{{\mathrm{HbO}}_{2}}$, $\varepsilon_{\mathrm{Hb}}$, and $\varepsilon_{\text{melanin}}$ are the molar extinction coefficients of oxygenated hemoglobin, deoxygenated hemoglobin, and melanin, respectively, and $C_{{\mathrm{HbO}}_{2}}$, $C_{\mathrm{Hb}}$, and $C_{\text{melanin}}$ are the concentrations of oxygenated hemoglobin, deoxygenated hemoglobin, and melanin, respectively.

In pixel-wise fitting,^8^ the squared error

$$\text{error}=\overset{3}{\underset{i=1}{\sum }}[({\mathrm{MTF}}_{\mathrm{AC}}(\lambda_{i})-{\mathrm{mtf}}_{\mathrm{AC}}(\lambda_{i}){)}^{2}+({\mathrm{MTF}}_{\mathrm{DC}}(\lambda_{i})-{\mathrm{mtf}}_{\mathrm{DC}}(\lambda_{i}){)}^{2}]$$ (10)

is minimized where i=1, 2, and 3 represent the three red (R), green (G), and blue (B) wavelengths of the light source, ${\mathrm{MTF}}_{\text{DC}}$ and ${\mathrm{MTF}}_{\mathrm{AC}}$ are the values measured in the experiment, and ${\mathrm{mtf}}_{\mathrm{DC}}$ and ${\mathrm{mtf}}_{\mathrm{AC}}$ are the theoretical values at spatial frequencies 0 and $f_{x}$ for the equivalent homogeneous turbid medium. Grouping MTF into multiple clusters by k-means is used in the perturbative SFDI method to speed up the recovery.^23^ Skin optical, structural, and physiological parameters such as the THb, ${\mathrm{StO}}_{2}$, $\mu_{s}^{\prime }$, scattering power (b), melanin content, surface roughness (α), and epidermal thickness (h) are then obtained. Although pixel-wise fitting and p-SFDI can recover a rich set of skin properties, their inversion speed is slow and does not meet the demands of clinical applications. In contrast, deep-learning–enabled SFDI-net significantly improves image quality by effectively suppressing noise while preserving tissue structure without oversmoothing and achieves a real-time inversion speed [see Fig. 1(b)].

### 2.2 SFDI-net Architecture

**Fig. 2:**
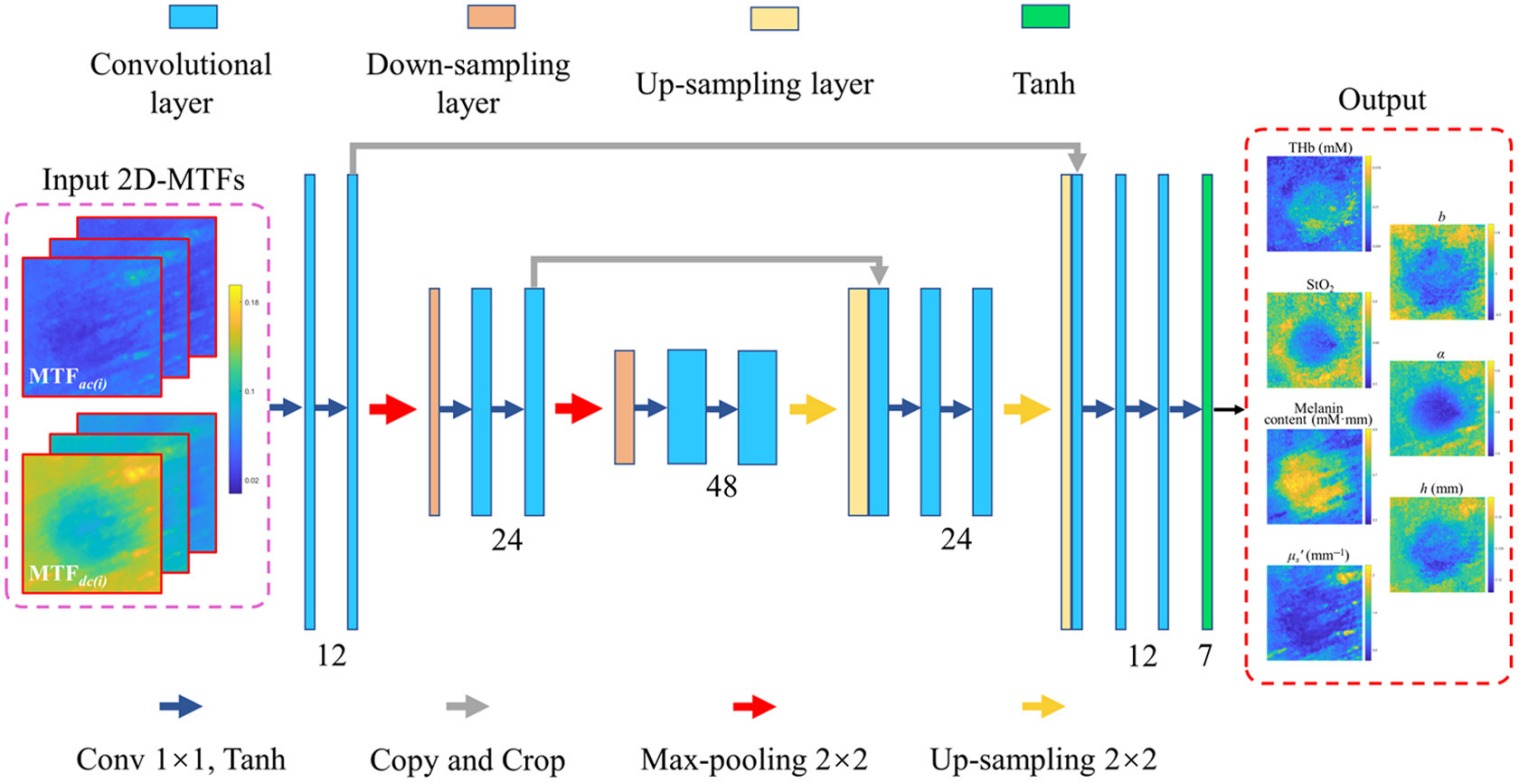
Architecture of SFDI-net comprises a contracting path with convolutional layers having 12, 24, and 48 channels and an expansive path with convolutional layers having 24 and 12 channels. The inputs are DC and AC MTF maps at three RGB wavelengths. The output predicts seven channel images of THb, ${\mathrm{StO}}_{2}$, melanin content, $\mu_{s}^{\prime }$, b, α, and h.

SFDI-net is built using a convolutional neural network (CNN) architecture inspired by the U-Net framework suitable for image-to-image translation.^32^^,^^33^ SFDI-net comprises a contracting path for feature extraction and an expansive path for precise localization (see Fig. 2). The network input has a shape of n×n×6 for DC and AC MTF images (size n×n) from three RGB channels. The contracting path adheres to a conventional CNN layout, repetitively applying two 1×1 convolutional kernels with zero padding and a stride of 2, each succeeded by a tanh activation function. Downsampling is achieved using 2×2  max pooling blocks with a stride of 2 twice, halving the feature map dimensions with each iteration. Concurrently, the feature channel count doubles at each downsampling step, with convolutional layers having 12, 24, and 48 channels. The expansive path undergoes two upsampling operations, doubling the feature map dimensions each time. Each step encompasses a 2×2 upsampling convolutional layer with a stride of 2, halving the feature channel count, concatenating with the corresponding feature map from the contracting path, followed by the application of two 1×1 convolutional kernels with zero padding and a stride of 2, and then tanh activation. The convolutional layers in this path have 24 and 12 channels, respectively. The network’s output layer employs a 1×1 convolutional kernel with zero padding and a stride of 2, subsequently normalizing the prediction between −1 and 1 using tanh activation. Tanh rather than rectified linear unit (ReLU) activation was used due to its better performance. The number of convolutional layers is half that of the traditional U-Net architecture. This reduction in model complexity significantly increases the computational speed.

### 2.3 Data Collection

The illumination of sinusoidal fringe patterns from a digital micromirror device (DMD, DLP LightCrafter™ 4500, Texas Instruments, United States) was projected onto the specimen via a pellicle beam splitter. The visible light consists of RGB colors with wavelengths of 623, 540, and 460 nm. A CMOS camera (Point Gray Grasshop3 GS3-U3-51S5C) records backscattered light reflectance images of the sample. We set the spatial frequency $f_{x}=0.2\;{\mathrm{mm}}^{-1}$ with phases of 0, 120, and 240 deg for three-phase demodulation. Furthermore, calibration procedures were conducted to correct the nonlinearity and nonuniformity of the DMD projection.^8^^,^^23^ We also removed the cross-channel contamination caused by white light illumination on the color camera.^8^^,^^23^ These steps ensure that the absolute MTF is measured. All measurements were performed under normal incidence and detection conditions.

Twenty-six healthy volunteers (11 males and 15 females, 18 to 28 years old) were imaged under reactive hyperemia on the forearm for 7 min. The cuff pressure rapidly increased to 200 mmHg at 1.5 min following a baseline measurement. The cuff pressure was then released after 2 min and 40 s, followed by continuous monitoring for another 2 min and 50 s. Another healthy volunteer (female, 26 years old) also underwent the same experiment for dynamic quantitative imaging of forward reactive hyperemia. A second healthy subject (female, 21 years old) was imaged under low-frequency rhythmic respiration for 3 min. The subject exhaled and inhaled for 6 s each, resulting in a breathing frequency of five breaths per minute. The hemodynamic oscillations due to rhythmic respiration were extracted by removing a polynomial fit to the data.^12^ Images of melanin nevus on the forearms of four volunteers (three males and one female, 24 to 27 years old) were also obtained while maintaining a stable condition at rest during the measurement. The melanin nevus data from one of the four volunteers were reserved for validation (and not used in training). All study subjects were of Chinese ethnicity, and their skin was categorized as Fitzpatrick skin phototypes III and IV. Table 1 lists the range of physiological and optical parameters for the subjects. The study protocol was approved by the Ethics Committee of the Eye and Optometry Hospital of Wenzhou Medical University. Informed consent was obtained from all subjects before measurements were performed.

**Table 1:** Range of physiological and optical parameters for the subjects with Fitzpatrick skin phototypes III and IV.

	THb (mM)	${\mathrm{StO}}_{2}$	Melanin content (mM·mm)	Scattering power	Surface roughness	Epidermal thickness (mm)	$\mu_{a}$ (${\mathrm{mm}}^{-1}$)			$\mu_{s}^{\prime }$ (${\mathrm{mm}}^{-1}$)
							632 nm	540 nm	460 nm	540 nm
Range	0.0005	0.31	0.40	−1.50	0.10	0.05	0.01	0.02	0.05	0.29
	0.0249	0.97	1.50	1.50	1.50	0.19	0.33	0.70	1.00	2.37

### 2.4 Data Preprocessing

A total of 13,020 input–output pairs were obtained via pixel-wise fitting from forearm reactive hyperemia and melanin nevus measurements. This dataset included 10,920 pairs from forearm reactive hyperemia experiments on 26 volunteers with significant differences in skin color. Each subject performed a forearm reactive hyperemia experiment twice, acquiring 210 sets of pairs for each experiment. The remaining 2100 input–output pairs were randomly selected from the results of melanin nevus measurements. As the difference in melanin content between melanotic and normal human skin is significant, including both sets of training data is beneficial for avoiding overfitting.

For data processing, we cropped the experimentally derived 2D MTF to a size of 128×128×6. The values of the cropped MTFs were subsequently clustered into 15 distinct groups using k-means. We then randomly extracted 15 input–output pairs generated by pixel-wise fitting above to populate the respective clusters. Through the iterative application of this methodology, we successfully assembled a total of 1248 such pairs. This data processing approach effectively mitigates the time required for the network to learn the underlying distribution pattern. Furthermore, we obtained an additional 200 such pairs using the same method for the testing dataset.

### 2.5 Training Details

SFDI-net was implemented in TensorFlow’s Keras framework in Python. The Adam optimizer was used to optimize the CNN model. The training initial learning rate was set to 0.00055, and the batch size was set to 32. The loss function, consisting of the mean squared error and total variance (TV) loss, is given by

L(y^,y)=17n2∑i,j,k(y^i,j,k−yi,j,k)2+17n2∑i,j,kwk[|(y^i,j+1,k−y^i,j,k)|+|(y^i+1,j,k−y^i,j,k)|], (11)

where y and y^ denote the ground truth and output images, respectively; ${\hat{y}}_{i,j+1}$ and ${\hat{y}}_{i+1,j}$ are the adjacent pixels of ${\hat{y}}_{i,j}$; i and j are the vertical and horizontal coordinates, respectively; and $w_{k}$ is the TV weight for k=1,2,…,7. The total variation loss can suppress noise and avoid overpixelation of the generated images.^34^^,^^35^ The weights ($w_{k}$) are set as follows: THb: $1.0\times 10^{-7}$; ${\mathrm{StO}}_{2}$: $2.5\times 10^{-7}$; melanin content: $5.0\times 10^{-8}$; $\mu_{s}^{\prime }$: $1.0\times 10^{-8}$; b: $1.0\times 10^{-7}$; α: $1.0\times 10^{-8}$; and h: $2.0\times 10^{-6}$, accounting for the different influences of noise on the respective outputs and their spatial distribution characteristics.

A total of 1248 pairs of MTFs with dimensions of 128×128×6, along with optical, structural, and physiological parameters of 128×128×7, were split 90%:10% randomly into training and validation sets. Cross-validation is used to optimize and assess the model performance.^36^ The network was trained using an NVIDIA GTX 1080Ti with 100 epochs.

In addition, the coefficient of determination, $R^{2}$, is computed as one metric to evaluate the predictive performance of the SFDI-net. It quantifies the proportion of explained variance and is formally defined as follows:

R2=1−∑i,j,k(yi,j,k−y^i,j,k)2∑i,j,k(yi,j,k−y¯i,j,k)2, (12)

where $\overset{¯}{y}$ denotes the mean value. A higher $R^{2}$ value closer to one indicates closer agreement between the model’s predictions and the ground truth.

## 3 Results

### 3.1 Training and Validation Loss

**Fig. 3:**
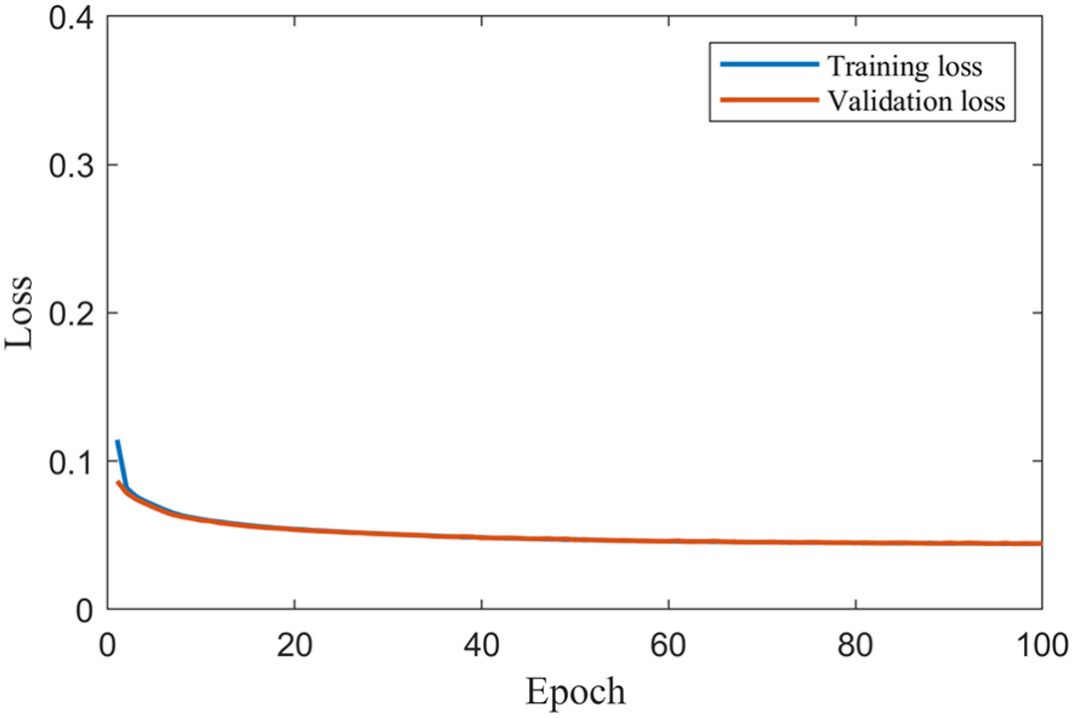
Training and validation loss.

Figure 3 shows the training and validation loss curves. The training and validation loss decreased to 0.05 at epoch 25, and no further reduction was observed with increasing epochs.

### 3.2 Network $R^{2}$

The $R^{2}$ values for the SFDI-net on the training, validation, and testing datasets are 0.698, 0.700, and 0.694, respectively. This suggests that SFDI-net has no overfitting and can generalize to new, unseen data. The inclusion of TV loss in SFDI-net enhances the robustness but sacrifices the degree of prediction accuracy measured by $R^{2}$. The $R^{2}$ values of the training, validation, and testing datasets increase to 0.854, 0.852, and 0.865, respectively, if the TV loss is removed from the SFDI-net model.

### 3.3 Validation of SFDI-net on Fast 2D Mapping of Optical, Structural, and Physiological Parameters from Simulated Data

**Fig. 4:**
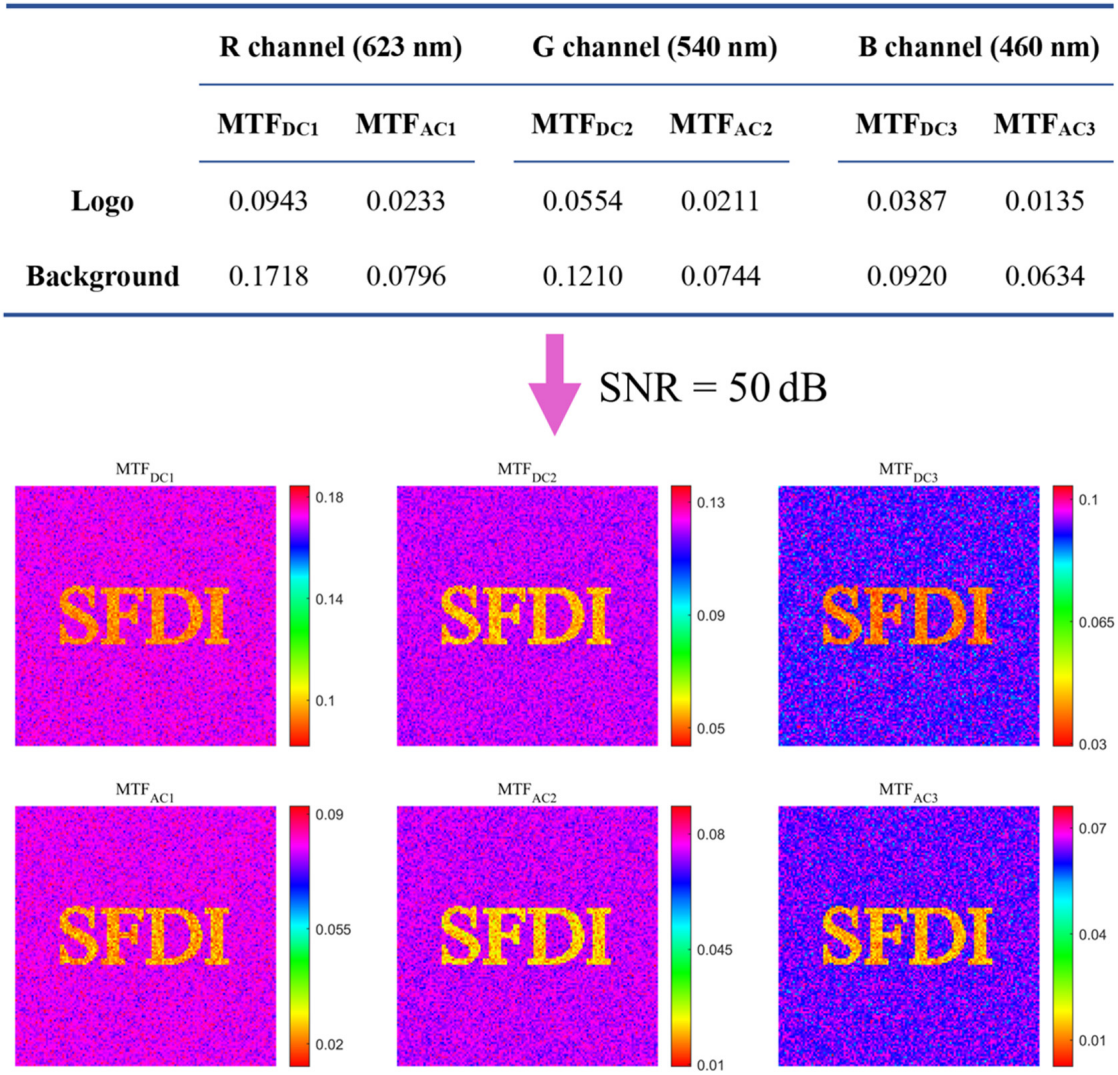
Simulated MTF maps at SNR=50  dB.

We then validate SFDI-net via simulations. Two sets of representative MTFs at wavelengths of 460, 540, and 623 nm and a spatial frequency of $0.2\;{\mathrm{mm}}^{-1}$ were used to construct a digital tissue phantom (see Fig. 4). The ground truth for the optical, structural, and physiological parameters was determined according to the analytical two-layer skin model. Gaussian noise was added to the resulting 128×128×6 MTF images to achieve a signal-to-noise ratio (SNR) of 50 dB for each channel. The parameter k for k-means clustering in p-SFDI is set to 20.

**Fig. 5:**
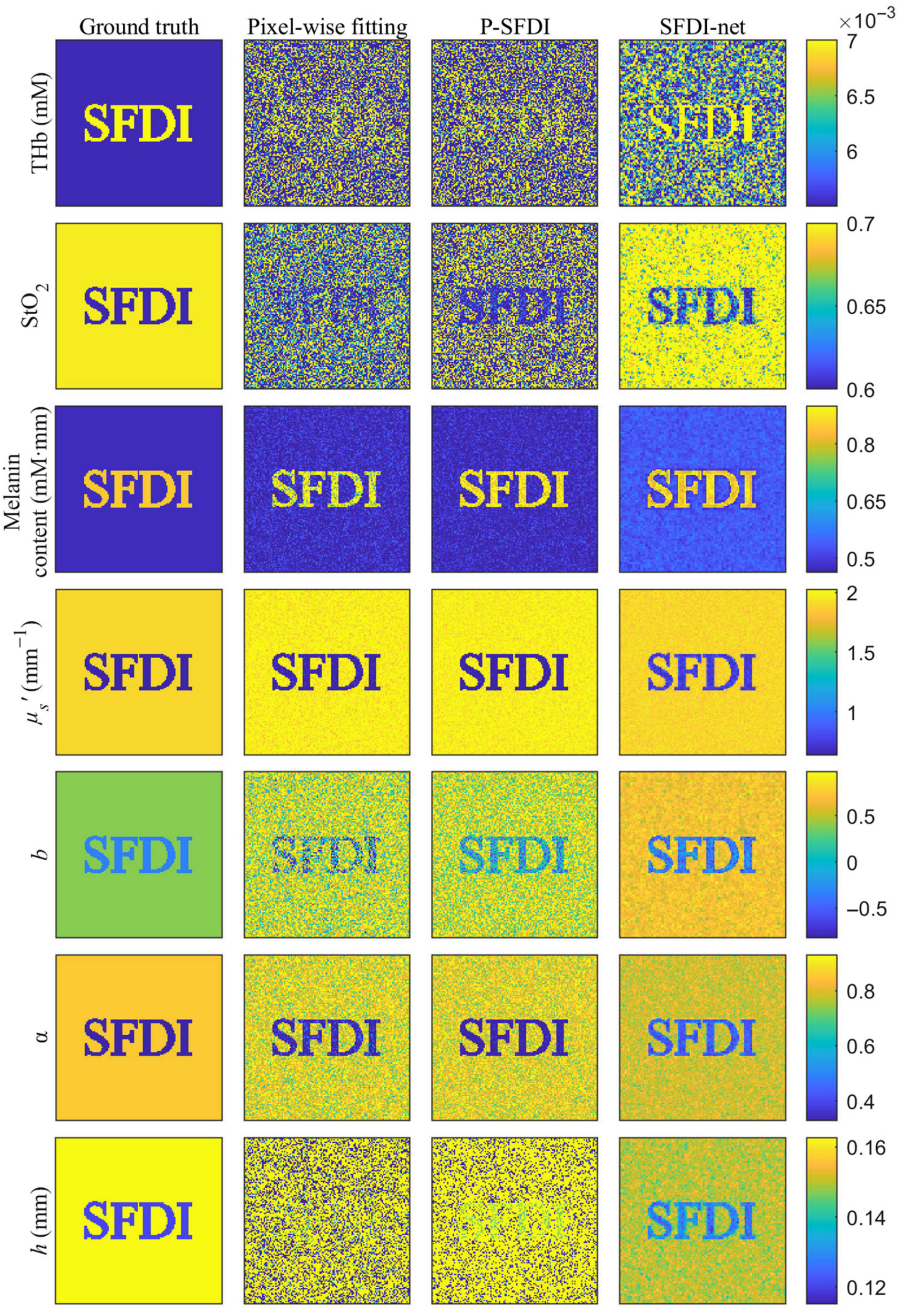
Optical, structural, and physiological parameters recovered by pixel-wise inversion, p-SFDI, and SFDI-net compared with the ground truth.

The accuracy of the optical, structural, and physiological parameters recovered by the pixel-by-pixel least squares fitting, p-SFDI, and SFDI-net methods are compared in Fig. 5. Notably, the pixel-wise fitting exhibited varying degrees of susceptibility to noise, with parameters such as melanin content, $\mu_{s}^{\prime }$, b, and α maintaining good image quality, whereas THb, ${\mathrm{StO}}_{2}$, and h exhibited substantial noise-induced degradation, rendering the logo indiscernible. p-SFDI improved the clarity of the logo in the ${\mathrm{StO}}_{2}$ and b images, albeit with a limited restoration of THb and h. In contrast, all images reconstructed using SFDI-net exhibited remarkable clarity, displaying significantly reduced noise and enhanced legibility.

Table 2 shows the mean absolute percentage error (MAPE) and structural similarity index measure (SSIM) of images for the logo region containing the “SFDI,” the background region, and the whole image. SFDI-net outperforms pixel-wise fitting in terms of the recoveries of THb, ${\mathrm{StO}}_{2}$, b, and h with the lowest MAPE. The MAPE for melanin, $\mu_{s}^{\prime }$, and α recovered by the SFDI-net are similar to those of other methods. The $\mu_{s}^{\prime }$ and α retrieved by SFDI-net have increased errors in the logo region but smaller errors in the background region than other approaches. One potential factor contributing to this phenomenon is the incorporation of the TV loss within SFDI-net, which, while enhancing denoising capabilities, may introduce a trade-off in accuracy for sharp structures.

**Table 2:** MAPE and structural similarity of optical, structural, and physiological parameters recovered by pixel-wise inversion, p-SFDI, and SFDI-net.

	Method	THb (mM)	${\mathrm{StO}}_{2}$	$C_{\text{melanin}}$ (mM·mm)	$\mu_{s}^{\prime }$ (${\mathrm{mm}}^{-1}$)	b	α	h (mm)
MAPE (STD) (logo)	Pixel-wise fitting	31.93% (0.00280)	16.45% (0.138)	12.31% (0.127)	9.27% (0.068)	192.8% (0.757)	32.15% (0.093)	35.28% (0.036)
	p-SFDI	25.25% (0.00216)	2.61% (0.015)	5.50% (0.055)	6.74% (0.043)	72.45% (0.323)	18.02% (0.039)	39.37% (0.013)
	SFDI-net	28.54% (0.00209)	4.19% (0.029)	4.59% (0.048)	22.95% (0.068)	40.02% (0.168)	72.12% (0.047)	7.44% (0.005)
MAPE (STD) (background)	Pixel-wise fitting	33.80% (0.00234)	18.13% (0.143)	7.23% (0.044)	6.04% (0.094)	102.1% (0.418)	8.75% (0.091)	21.87% (0.051)
	p-SFDI	33.26% (0.00235)	13.61% (0.111)	5.82% (0.036)	5.53% (0.074)	87.97% (0.300)	7.04% (0.073)	19.20% (0.046)
	SFDI-net	17.01% (0.00101)	4.79% (0.038)	12.15% (0.020)	1.71% (0.041)	77.21% (0.099)	7.63% (0.037)	18.21% (0.003)
SSIM (whole image)	Pixel-wise fitting	0.993	0.060	0.433	0.264	0.020	0.244	0.270
	p-SFDI	0.993	0.108	0.523	0.308	0.031	0.297	0.325
	SFDI-net	0.995	0.481	0.793	0.502	0.179	0.501	0.943

The SSIM of images evaluates the similarity between the restored images and the ground truth. An SSIM closer to one signifies greater similarity to the ground truth. The SFDI-net consistently demonstrated much greater structural similarity than did the p-SFDI and pixel-wise inversion methods. This superiority was particularly pronounced for parameters such as melanin content and h. Therefore, the SFDI-net more accurately delineated the spatial structure.

### 3.4 Comparison of 2D Quantitative Imaging of Melanin Nevi by Pixel-Wise Fitting, p-SFDI, and SFDI-net

**Fig. 6:**
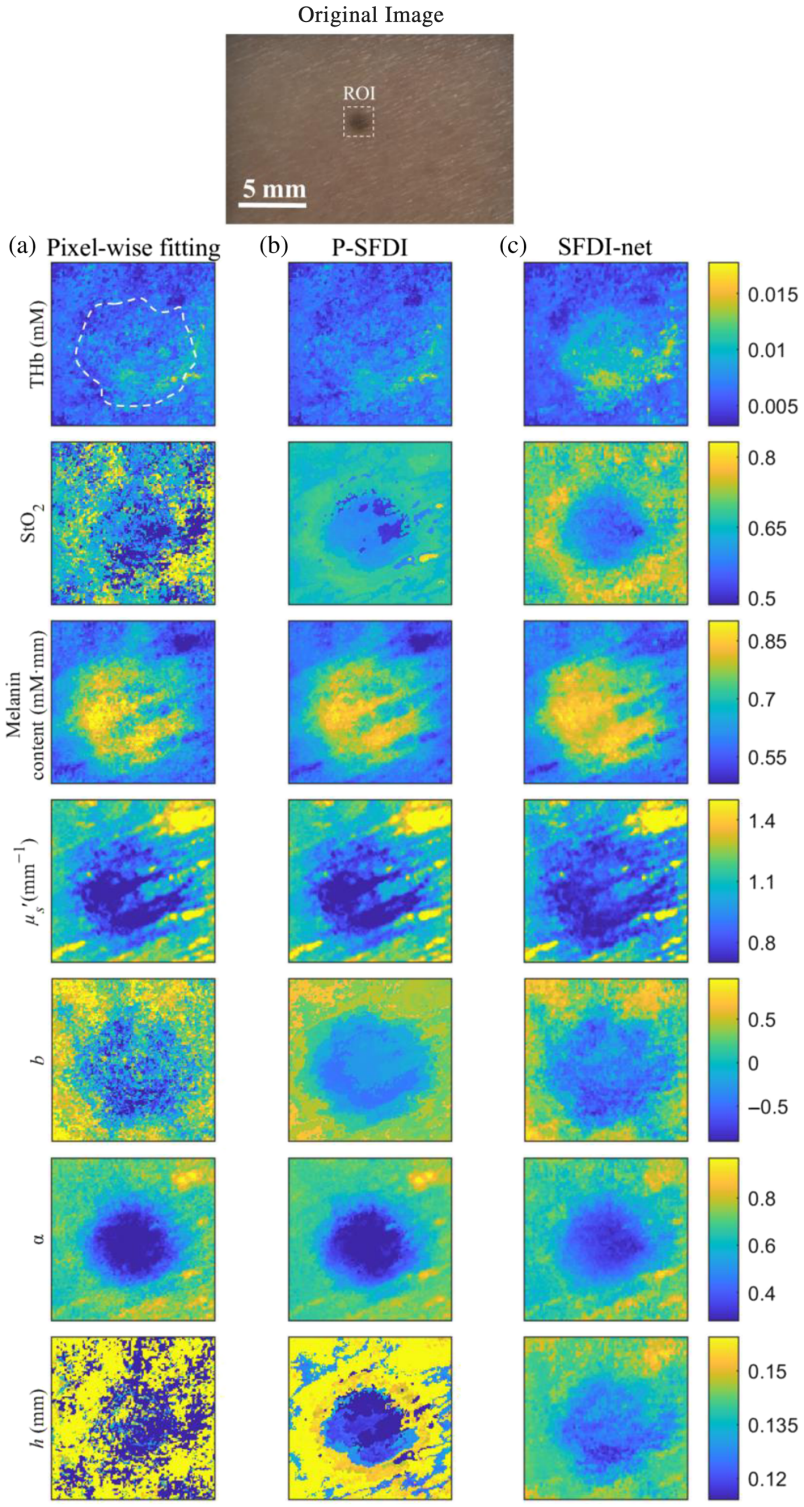
Two-dimensional maps of THb, ${\mathrm{StO}}_{2}$, melanin content, $\mu_{s}^{\prime }$, b, α, and h recovered by (a) pixel-wise inversion, (b) p-SFDI, and (c) SFDI-net for the region of interest (ROI) (the white dashed box) within the image field of view. The white dashed line in panel (a) marks the nevus region.

Figure 6 displays the 2D maps of the THb, ${\mathrm{StO}}_{2}$, melanin content, $\mu_{s}^{\prime }$, b, α, and h of melanocytic nevi from the forearm of one volunteer recovered by pixel-wise fitting, p-SFDI, and SFDI-net. Compared with pixel-wise fitting, p-SFDI and SFDI-net perform significantly better in recovering the nevus morphology because of their superior measurement noise suppression and provide a clear contrast between the nevus region and the surrounding skin. In addition, the SFDI-net revealed skin texture and different THb and ${\mathrm{StO}}_{2}$ levels between nevus and normal skin more clearly than did the p-SFDI. These observations indicate that SFDI-net can suppress noise while simultaneously preserving tissue structure (avoiding oversmoothing). The means and standard deviations of the seven parameters inverted by the three methods are shown in Table 3 for the nevus region and the surrounding tissue. Overall, the average optical, structural, and physiological parameters retrieved by the three methods agreed with each other. The contrast between the nevus region and the surrounding tissue is best revealed by SFDI-net among the three methods, with higher THb, lower ${\mathrm{StO}}_{2}$, and higher melanin content in the nevus region, which agrees with the findings in Ref. 37. The lower reduced scattering coefficient associated with higher melanin content in Fig. 6 has also been observed in other recent studies,^37^^–^^39^ attributed to the confounding effects of light absorption by melanin, which limits the probing depth and results in fewer photons reaching the collagen and elastin matrix in the dermis.^40^^,^^41^

**Table 3:** Average and standard deviation of optical, structural, and physiological parameters recovered by pixel-wise inversion, p-SFDI, and SFDI-net for the nevus and surrounding tissue.

	Method	THb (mM)	${\mathrm{StO}}_{2}$	$C_{\text{melanin}}$ (mM·mm)	$\mu_{s}^{\prime }$ (${\mathrm{mm}}^{-1}$)	b	α	h (mm)
Mean (STD) (nevus)	Pixel-wise fitting	0.00697 (0.00180)	0.601 (0.116)	0.761 (0.068)	0.838 (0.158)	−0.213 (0.420)	0.453 (0.171)	0.130 (0.042)
	p-SFDI	0.00702 (0.00160)	0.631 (0.053)	0.759 (0.053)	0.835 (0.149)	−0.217 (0.177)	0.443 (0.157)	0.134 (0.025)
	SFDI-net	0.0721 (0.00222)	0.648 (0.069)	0.781 (0.052)	0.820 (0.112)	−0.336 (0.208)	0.493 (0.117)	0.130 (0.006)
Mean (STD) (surrounding tissue)	Pixel-wise fitting	0.00652 (0.00163)	0.643 (0.120)	0.598 (0.051)	1.174 (0.216)	0.373 (0.359)	0.696 (0.075)	0.144 (0.046)
	p-SFDI	0.00650 (0.00154)	0.663 (0.023)	0.600 (0.048)	1.173 (0.209)	0.362 (0.182)	0.695 (0.059)	0.156 (0.018)
	SFDI-net	0.0635 (0.00171)	0.705 (0.038)	0.610 (0.049)	1.085 (0.231)	0.264 (0.268)	0.676 (0.074)	0.141 (0.005)

### 3.5 Dynamic Quantitative Imaging of Forearm Reactive Hyperemia

**Fig. 7:**
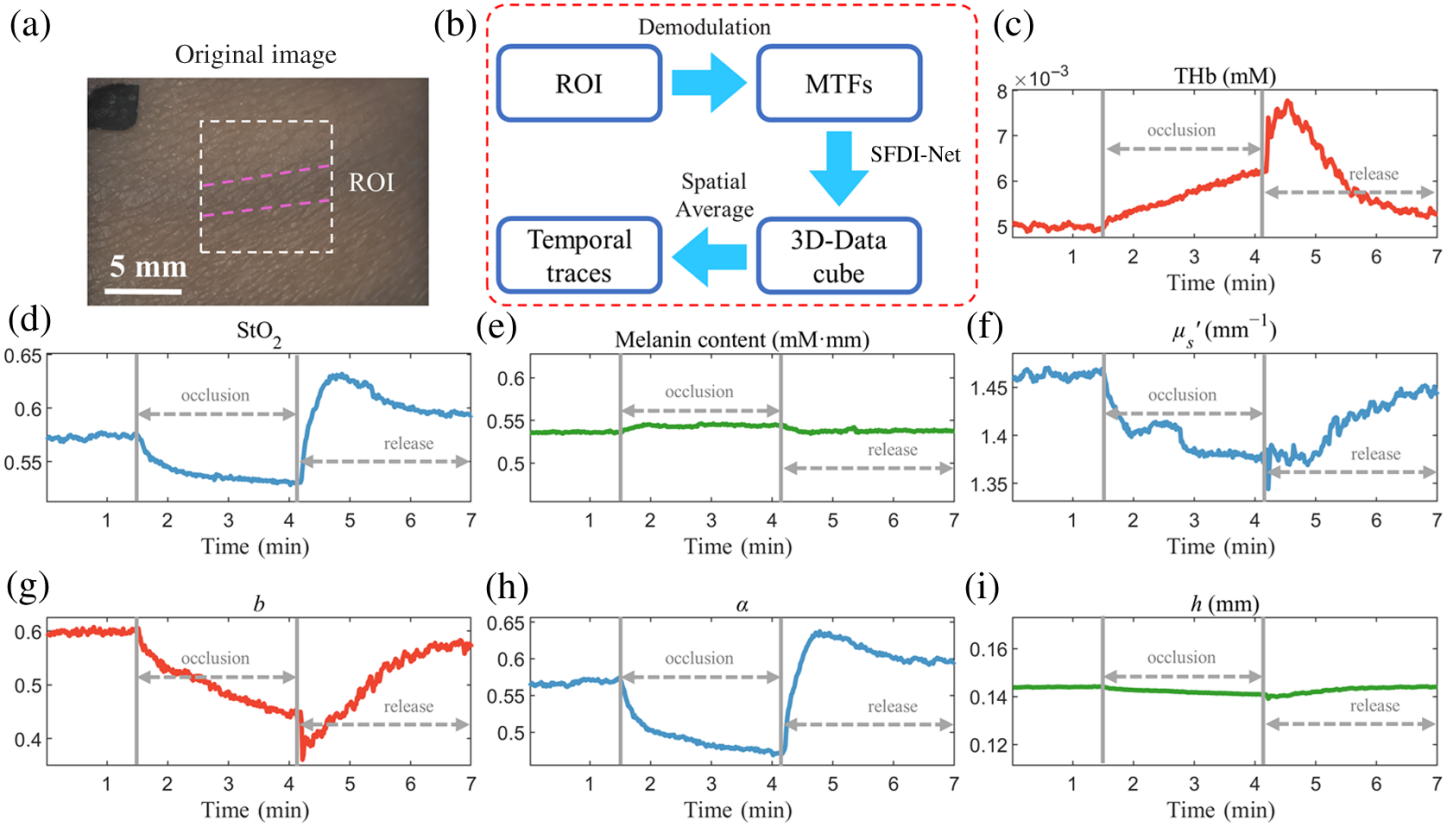
Forearm reactive hyperemia (multimedia available online). (a) Photo of the forearm being imaged. The white dashed box indicates the ROI. (b) Flowchart for generating temporal traces. (c)–(i) Temporal traces of THb, ${\mathrm{StO}}_{2}$, melanin concentration, reduced scattering coefficient, scattering power, surface roughness, and epidermal thickness of the ROI during forearm reactive hyperemia.

Figure 7 (multimedia available online) displays the mean alterations in optical, structural, and physiological parameters within the ROI during forearm reactive hyperemia. Notably, Figs. 7(c) and 7(d) reveal that during the period of cuff occlusion, circulatory stasis occurs within the forearm vasculature, leading to minimal fluctuations in hemoglobin content despite ongoing oxygen consumption by the tissue when ${\mathrm{StO}}_{2}$ decreases. Upon the release of the occlusion, a substantial influx of fresh blood is observed within the forearm’s vasculature, culminating in an increase in THb and ${\mathrm{StO}}_{2}$ levels. These values eventually revert to their baseline levels. Melanin content (0.548 mM·mm) and epidermal thickness h (0.142 mm) were stable across the entire duration of forearm reactive hyperemia, consistent with previous studies.^8^^,^^23^ A video of the spatiotemporal dynamics of the forearm under reactive hyperemia (7 min long, at 1 fps) is shown in Video 1. The video was accelerated 30 times for easy viewing.

**Fig. 8:**
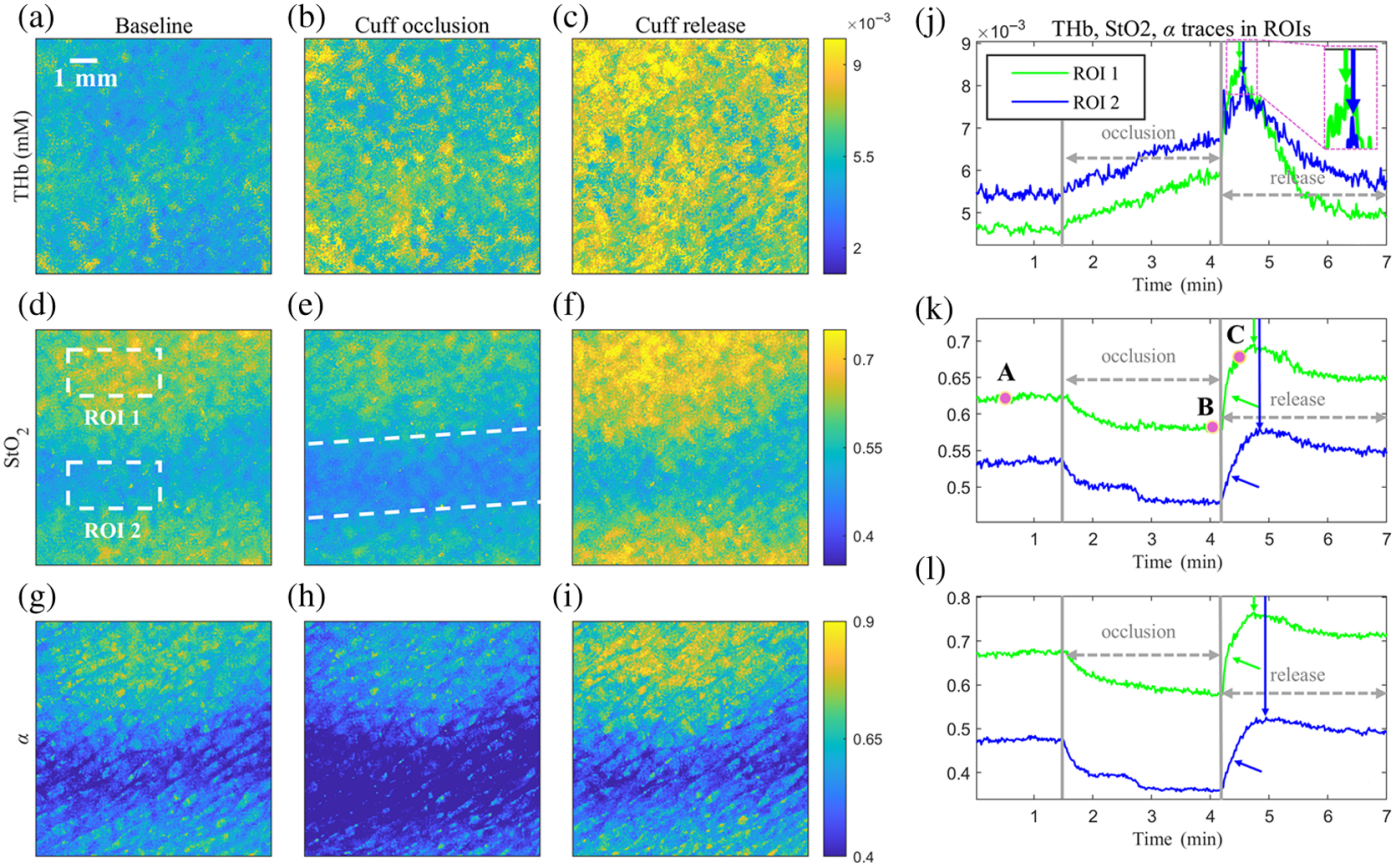
2D images of THb (a)–(c), ${\mathrm{StO}}_{2}$ (d)–(f), and α (g)–(i) captured at times A (baseline), B (immediately before), and C (immediately after cuff release). The temporal traces of THb, ${\mathrm{StO}}_{2}$, and α are shown in (j)–(l) for ROIs 1 and 2, marked by dashed white rectangles. The dashed white lines outline the venous area. The capillary area rises faster than the venous region after cuff release [see arrows in panels (k) and (l)].

Figure 8 illustrates spatiotemporal variations in THb, ${\mathrm{StO}}_{2}$, and α before and after cuff release, alongside the average temporal trajectories within two distinct ROIs, one encompassing normal skin tissue and the other over the venous region. A lower ${\mathrm{StO}}_{2}$ is observed within the venous area than in other regions, consistent with physiological expectations. Following tissue gas exchange at the capillary level, blood returns to the venous compartment, characterized by higher levels of deoxygenated hemoglobin and a lower ${\mathrm{StO}}_{2}$ within the venous territory. Furthermore, Figs. 8(g)–8(i) show significant divergences in α between the normal skin region and the venous domain, indicating differences in the refractive index disparities at the interfacial boundary and the skin surface roughness experienced by diffusive light over the two regions. Temporal variations in THb, ${\mathrm{StO}}_{2}$, and α averaged over the two ROIs, as delineated in Figs. 8(j)–8(l), show that the temporal dynamics within the venous region lag those in the normal region. This observation aligns with the physiological expectation that blood traverses capillaries before re-entering venous circulation.

The relative temporal delays are different for the three parameters following cuff release. THb attains its peak at the earliest instance, accompanied by a delay of 3 s between the two ROIs. ${\mathrm{StO}}_{2}$ follows suit with a 5-s delay, whereas alterations in α manifest the largest temporal delay of 12 s between the two ROIs. This suggests that the response in THb is the fastest. In contrast, cellular structures require additional time to respond to this hemodynamic variation, resulting in the largest lag in α.

### 3.6 Dynamic Quantitative Imaging of the Skin Under Rhythmic Respiration

**Fig. 9:**
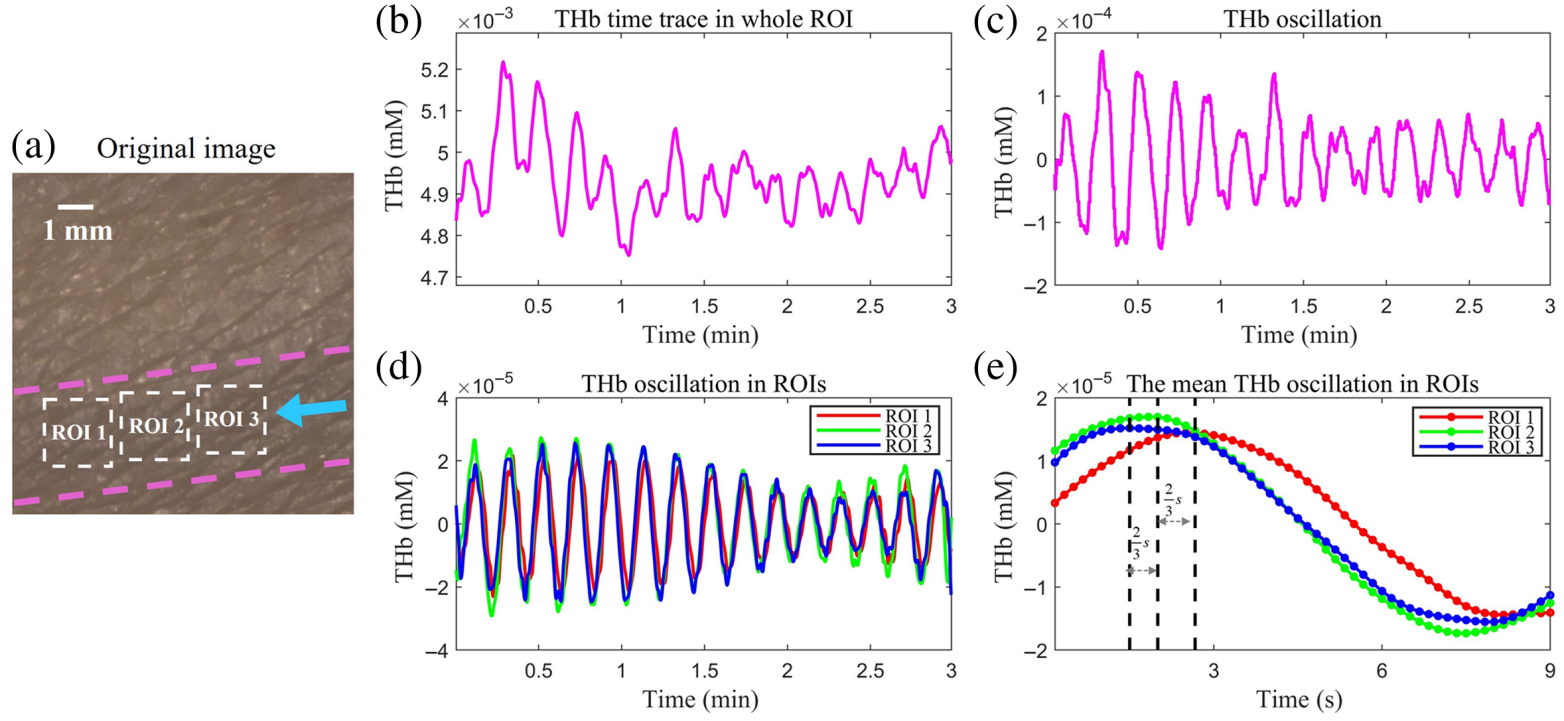
Spatiotemporal cutaneous hemodynamics under rhythmic respiration (multimedia available online). (a) Photo of the forearm being imaged. The white dashed rectangles mark the ROIs. The blue arrow points to the direction of blood flow inside the venous region, delineated by purple dashed lines. (b) and (c) Temporal trace of THb of the whole field of view and its detrended THb oscillations due to paced breathing. (d) and (e) THb oscillations of ROIs 1, 2, and 3 and their average THb oscillation pattern.

Coupling between the respiratory and cardiovascular systems, known as “cardiorespiratory coupling,” encapsulates a wealth of physiological and pathological insights into the overall health status of the organism.^42^^,^^43^ The study of hemodynamic alterations induced by low-frequency rhythmic respiration has attracted significant attention.^12^^,^^44^ Figure 9 (multimedia available online) shows spatiotemporal cutaneous hemodynamics under rhythmic respiration for one healthy subject at five cycles per minute. A video of the spatiotemporal dynamics of the forearm under rhythmic respiration (24 s long, at 3 fps) is shown in Video 2.

Figure 9(b) displays the time trace for THb over the field of view. The detrended THb time traces reveal discernible periodic oscillations in THb levels at the respiration rate over the entire FOV and individual ROIs [see Figs. 9(c) and 9(d)]. Furthermore, the oscillations in ROI 1 lagged behind ROI 2, and those in ROI 2 lagged behind ROI 3, which coincided with the direction of blood flow. Figure 9(e) shows the mean THb patterns for ROIs 1, 2, and 3 obtained by averaging the THb oscillations across each cycle in Fig. 9(d). The phase lags are 0.4 and 0.6 s for ROIs 2 and 3, respectively. Furthermore, the center-to-center distance among adjacent ROIs was 1.92 mm. Subsequently, the respiratory wave velocity in the venous area is estimated to be 3.8  mm/s. The blood flow inside vessels 0.1 mm or smaller (capillaries and venules) is quasistatic. The wave speed for such a small Womersley number pulsatile flow of angular frequency ω is proportional to √ω.^22^ The observed respiratory wave speed falls within a reasonable range inferred from the blood flow speed of ∼12  mm/s, which is driven by the heartbeat inside the venous region.^45^

## 4 Discussion and Conclusion

We have presented deep-learning–enabled spatial frequency domain imaging, termed SFDI-net, for imaging the spatiotemporal dynamics of skin physiology. It enables real-time two-dimensional mapping of skin optical, structural, and physiological parameters, including cutaneous hemoglobin concentration, oxygen saturation, scattering properties, melanin content, surface roughness, and epidermal thickness, from the reflection of spatially modulated visible light. SFDI-net, built using the U-net architecture, incorporates total variance loss to mitigate measurement noise and pixelation artifacts and enhance the spatial structural fidelity of recovery. It is important to note that the stratified skin structure is accounted for in SFDI-net by adopting a bilayer model, unlike many reported SFDI studies. Ignoring the layered structure can lead to significant discrepancies in the estimation of total hemoglobin concentration, oxygen saturation, and reduced scattering coefficient.^8^^,^^46^ The extra parameters obtained by SFDI-net are informative regarding skin structure and function as well and can, for example, aid the diagnosis of skin conditions related to aging, aesthetic procedures, and diseases.^3^^,^^7^^,^^47^

The SFDI-net is trained on the measured skin reflectance of flat surfaces under normal incidence and detection geometries. Furthermore, SFDI-net is applicable to SFDI measurements on nonflat surfaces and oblique incidence and detection geometries as long as the reflectance is corrected in advance according to the surface profile and distance estimation.^48^^,^^49^ An assemble procedure from measurements on 30 volunteers was used to generate the training, validation (n=1248 for training plus validation datasets), and testing (n=200) datasets. These datasets have high diversity and distinct data (pairs of 128×128×6 MTF and 128×128×7 optical, structure, and physiological parameter maps). SFDI-net performs equally well across the training, validation, and testing datasets, indicating that SFDI-net can generalize to new, unseen data.

Validation by numerical simulations shows that the SFDI-net significantly improves image quality. SFDI-net outperforms pixel-wise fitting in the recovery of THb, ${\mathrm{StO}}_{2}$, b, and h with the lowest MAPE, whereas the MAPE for melanin, $\mu_{s}^{\prime }$, and α recovered by SFDI-net are similar to those of pixel-wise fitting and p-SFDI. We note that inside sharp structures, such as the logo region, the $\mu_{s}^{\prime }$ and α retrieved by SFDI-net have greater errors than those retrieved by other approaches, partly caused by the TV loss within SFDI-net, which, while enhancing denoising capabilities, may introduce a trade-off in accuracy for smaller structures. Nevertheless, the SFDI-net method consistently achieved significantly greater structural similarity than did p-SFDI and pixel-wise inversion and more accurately delineated the spatial structure. Similar outperformance of SFDI-net over pixel-wise fitting and p-SFDI has also been observed for imaging melanin nevi.

The SFDI-net has finally been applied to analyze reactive hyperemia and rhythmic respiration in the forearm, monitoring spatiotemporal variations in optical, structural, and physiological parameters. The intricate spatiotemporal behaviors, such as the increase in THb, ${\mathrm{StO}}_{2}$, and α in the venous area lagging behind that in the surrounding areas after cuff release and blood oscillations caused by rhythmic respiration inside the venous region, were visualized. These experimental results on simulated and live subject data never seen by the network before further demonstrate the generalizability of SFDI-net and its potential for offering insights into skin physiology.

However, this work is not without limitations. The skin of human subjects is limited to Fitzpatrick skin phototypes III and IV due to data availability. We supplemented human subject data with images of melanin nevi on the forearms for the training dataset to enhance the range of the applicability and reliability of the SFDI-net regarding skin tones. Consequently, the range of melanin concentration in the SFDI-net is expanded to between 3.4 and 12.7 mM, covering the Fitzpatrick skin phototypes III, IV, V, and a portion of VI. Nevertheless, the collection and testing of skins of other Fitzpatrick skin phototypes for the SFDI-net is warranted to expand the applicability of SFDI-net to all skin types and evaluate its performance in future studies.

The SFDI-net significantly increased the inversion speed. It takes 31.25 ms to recover 512×512 images of the seven optical, structural, and physiological parameters on an NVIDIA GTX 1080Ti graphics processing unit (GPU) at speeds 3,700,000 and 1300 times faster than pixel-wise fitting and p-SFDI, respectively. Different SFDI approaches, which use machine learning, have recently emerged, including random forest regression (RFR), deep residual network (DRN), and single snapshot imaging of optical properties-deep learning (SSOP-DL).^13^^,^^17^^,^^23^^,^^50^ Like other models such as DRN and SSOP-DL for similar input dimensions, SFDI-net offers comparable inversion speeds. SFDI-net, however, uniquely provides comprehensive tissue optical, structural, and physiological parameters for layered skin using spatially modulated RGB light at the camera frame rate (see Table 4).

**Table 4:** Input type, retrieved parameters, and inversion speed by different SFDI approaches.

Parameter	Method					
	Pixel-wise fitting	p-SFDI	RFR	DRN	SSOP-DL	Deep-learning–enabled SFDI-net
Processor (model)	CPU (Intel I9-7900X)	CPU (AMD R7-1700)	CPU (Intel I5-7500)	CPU (Intel i7-8700)	GPU (NVIDIA GTX 1080Ti)	GPU (NVIDIA GTX 1080Ti)
Image size (pixel)	512 × 512	100 × 100	1000 × 1000	540 × 720	1024 × 1024	512 × 512
Wavelengths (nm)	460, 540, 623	460, 540, and 623	665	685 and 851	665	460, 540, and 623
Spatial frequency (${\mathrm{mm}}^{-1}$)	0.2	0.2	0.2	0.05, 0.1, 0.2, and 0.4	0.2	0.2
Output	THb, ${\mathrm{StO}}_{2}$, melanin content, $\mu_{s}^{\prime }$, b, α, and h	THb, ${\mathrm{StO}}_{2}$, melanin content, $\mu_{s}^{\prime }$, b, α, and h	$\mu_{a}$, $\mu_{s}^{\prime }$	THb and ${\mathrm{StO}}_{2}$	$\mu_{a}$, $\mu_{s}^{\prime }$	THb, ${\mathrm{StO}}_{2}$, melanin content, $\mu_{s}^{\prime }$, b, α, and h
Inversion time (ms)	$∼1.15\times 10^{8}$	$∼4.0\times 10^{4}$	450	48.1	18	31.25

SFDI-net is currently implemented with visible structured light. This choice of wavelengths optimizes the sensitivity of the SFDI-net to epidermal and dermal properties, as visible structured light is mostly confined within the epidermal and dermis layers of the skin and is insensitive to the subcutaneous layer below it.^7^^,^^8^ The current imaging speed is 3 fps, which is limited by the time latency in synchronous communication between the digital micromirror device and the camera, as well as the cumulative time required for three-phase demodulation image acquisition. A frame rate exceeding 10 fps is anticipated to be achieved by employing faster DMD and CMOS devices and optimizing the SFDI-net implementation.

Spatial frequency domain imaging has unique advantages in offering real-time spatiotemporal tissue optical, structural, and physiological properties. Future efforts will focus on refining the experimental system and SFDI-net to further increase the imaging speed and accuracy. Such advancements are expected to provide clinicians with expedited insights into tissue hemodynamics, structure, and physiology, facilitating prompt identification of pathological conditions, clinical disease monitoring, therapeutic assessment, and timely medical interventions.

## 5 Appendix: Supplementary Video

Video 1. The spatiotemporal dynamics of the forearm during reactive hyperemia over a period of seven minutes. The 2D maps and average temporal traces for optical, structural, and physiological parameters are shown. The white dashed lines mark the venous region (MP4, 2.87 MB [URL: https://doi.org/10.1117/1.JBO.30.4.046008.s1]).

Video 2. The spatiotemporal THb oscillations under rhythmic breathing. Three ROIs are outlined by dashed white lines inside the venous region marked by black dash lines. The ROIs are 1.67×1.67  mm in size and spaced 0.25 mm apart (MP4, 1.42 MB [URL: https://doi.org/10.1117/1.JBO.30.4.046008.s2]).

## Supplementary Material

10.1117/1.JBO.30.4.046008.s1

10.1117/1.JBO.30.4.046008.s2

## Data Availability

All code and data used for this work are available at https://github.com/BiomedPhotonics/SFDI-net.
